# Paternal preconceptional diet enriched with n-3 polyunsaturated fatty acids affects offspring brain function in mice

**DOI:** 10.3389/fnut.2022.969848

**Published:** 2022-10-28

**Authors:** Muhan Li, Qiaoyu Shi, Xueyi Jiang, Xuanyi Liu, Wei Han, Xiuqin Fan, Ping Li, Kemin Qi

**Affiliations:** Laboratory of Nutrition and Development, Key Laboratory of Major Diseases in Children, Ministry of Education, Beijing Pediatric Research Institute, Beijing Children’s Hospital, Capital Medical University, National Center for Children’s Health, Beijing, China

**Keywords:** n-3 polyunsaturated fatty acids, paternal nutrition, offspring brain, gene imprinting, mouse

## Abstract

Recent studies demonstrate that paternal nutrition prior to conception may determine offspring development and health through epigenetic modification. This study aims to investigate the effects of paternal supplementation of n-3 polyunsaturated fatty acids (n-3 PUFAs) on the brain development and function, and associated gene imprinting in the offspring. Three to four-week-old male C57BL/6J mice (founder) were fed with an n-3 PUFA-deficient diet (n-3 D), and two n-3 PUFA supplementation diets – a normal n-3 PUFA content diet (n-3 N) and a high n-3 PUFA content diet (n-3 H) for 12 weeks. Then they were mated to 10-week-old virgin female C57BL/6J mice to generate the offspring. The results showed that paternal n-3 PUFA supplementation in preconception reduced the anxiety- and depressive-like behavior, and improved sociability, learning and memory in the offspring, along with increased synaptic number, upregulated expressions of neuron specific enolase, myelin basic protein, glial fibrillary acidic protein, brain-derived neurotrophic factor in the hippocampus and cerebral cortex, and altered expressions of genes associated with mitochondria biogenesis, fusion, fission and autophagy. Furthermore, with paternal n-3 PUFA supplementation, the expression of imprinted gene Snrpn was downregulated both in testes of the founder mice and their offspring, but upregulated in the cerebral cortex and hippocampus, with altered DNA methylation in its differentially methylated region. The data suggest that higher paternal intake of n-3 PUFAs in preconception may help to maintain optimal brain development and function in the offspring, and further raise the possibility of paternal nutritional intervention for mental health issues in subsequent generations.

## Introduction

N-3 polyunsaturated fatty acids (n-3 PUFAs) are paramount for human health, and their functional roles in cardiovascular system, brain development and function, immune response, allergy, and particular physiological states (e.g., pregnancy, prematurity, infancy), have been focused during the past decades (1, 2). N-3 PUFAs are components of cellular membranes and the precursors of several metabolites with different beneficial effects on cell membrane fluidity, neuronal growth and differentiation, intracellular signaling and gene expression, inflammation and oxidation (3, 4). However, the modern western diet has greatly reduced the intake of n-3 PUFAs with an increase of n-6 PUFAs, resulting in the ratio of n-6/n-3 PUFAs at 20–30:1 which is much higher than that at 1–2:1 in the Paleolithic diet (5, 6). Growing evidence suggests that this altered dietary n-6/n-3 PUFAs is closely associated with chronic non-communicable diseases, including cardiovascular diseases, diabetes, obesity, as well as neurodevelopmental diseases (e.g., autism, attention deficit and hyperactivity disorder, and schizophrenia) in children, and degenerative neurological diseases in the elderly, and that the ratio of n-6 to n-3 PUFAs in diets at 1–2:1 should be the target ratio for health (7, 8).

The fetal programming hypothesis and thereafter the development origins of health and disease hypothesis indicate that nutrition and other environmental factors in early life determine the offspring phenotype and health in later life (9, 10). Both animal and human studies have demonstrated that maternal dietary n-3 PUFA deficiency during pregnancy and lactation impairs learning and memory in adult offspring (11, 12), and a high level of seafood intake or supplementation of docosahexaenoic acid (DHA, C22:6n-3) during pregnancy and lactation can effectively improve the psychological, language learning and intellectual development levels in early childhood (13, 14), although some inconsistent findings exist owing to multiple factors, such as differences in body n-3 PUFA baseline, quantity and duration of supplementation, interference from harmful chemicals or substances in seafood, and/or micronutrient (iron, iodine, zinc, etc.) deficiency (15–20).

During the past decade, a substantial number of studies have strengthened the paternal origins of health and disease paradigm, which stresses the need for more research on the role of the father in the transmission of acquired environmental messages from his environment to his offspring (21, 22). Mammalian spermatozoa are rich in PUFAs, particularly DHA, which are important for spermatogenesis with higher sperm motility and concentration, and normal morphology (23, 24). The reduction of n-3 PUFA intake in modern population leads to obstacles to spermatogenesis and maturation, resulting in the decline of male fertility, which has become a global problem in reproduction (25, 26). However, there is lack of relevant research on whether paternal n-3 PUFA status in preconception impacts on offspring development and health, including brain development and function.

The mechanisms through which parental nutrition determines offspring health have been extensively investigated, but they are still not completely understood. The contribution of maternal n-3 PUFAs to offspring brain development has been considered to be associated with several pathways, including enhancement of prenatal and postnatal DHA accretion in offspring brain, and epigenetic and non-epigenetic regulation on the expression of genes associated with neuronal growth and differentiation, protection against neuroinflammation, oxidation and apoptosis, etc. (4, 27, 28). Additionally, maternal feeding of DHA exerts preventive effects on prenatal stress-induced brain dysfunction through modulating metabolism of mitochondria (29), which plays a decisive role in brain development by providing energy for cell proliferation and differentiation, and synaptogenesis (30, 31). Being different from the direct interaction between the mother and offspring by nutrient exchange during prenatal and postnatal periods, the sperm- and seminal plasma-specific mechanisms connect paternal nutrition with the offspring development and health, as well as the maternal health (32). Gene imprinting, one class of epigenetics, is particularly relevant to early life and transgenerational effects since imprints are established in the germline, maintained during the preimplantation reprogramming phase, and then passed on through the somatic cell lineages impacting on genome function and gene expression (33, 34). Imprints are particularly promising candidates in brain research as they are known to be important for neurogenesis, brain function and behavior (33–36).

Therefore, we hypothesized that paternal higher n-3 PUFA intake in preconception might produce positive influences on brain development and function in the offspring through altering mitochondria metabolism and associated gene imprinting. In this study, using a mouse model that was received feeding intervention, the impact of preconception n-3 PUFA status in the father on the brain function (anxiety-like behaviors, depression-like behaviors, and memory) and histological changes were determined in the offspring. Furthermore, changes in offspring brain mitochondria metabolism, and expressions of imprinted genes associated with brain development in the testis and brain were investigated.

## Materials and methods

### Diets

Three types of diets with n-3 PUFA deficiency (n-3 D), normal n-3 PUFA content (n-3 N), or high n-3 PUFA content (n-3 H) were designed and manufactured by modifying the oil type in the AIN-93G diet as our previously published (37). The lard oil and sunflower oil were added in the n-3 D diet to produce n-3 PUFA deficiency with an n-6/n-3 PUFA ratio at 47.2:1; whereas the flaxseed oil and fish oil mixed with the lard oil and sunflower oil were added to the n-3 N and n-3 H diets to yield two different n-6/n-3 PUFA ratios at 4.3:1 and 1.5:1, respectively, which represent the current recommendation (4–10:1) and the dietary ratio for our ancestors, containing both very long-chain n-3 PUFAs, eicosapentaenoic acid (EPA; C20:5n-3), DHA and their precursor α-linolenic acid (ALA, C18:3n-3) (5–7). The AIN-93G growing diet and AIN-93M mature diet were used for maternal feeding during pregnancy and lactation, and offspring pup’s feeding after weaning, respectively. Details for the diet formula and fatty acid compositions are shown in Table 1. All the diets were prepared by the Beijing Huafukang Bioscience Co. Inc. (Beijing, China) and were sterilized with γ-irradiation 25 kGy and stored at −20°C before use.

**TABLE 1:** The ingredient compositions and fatty acid profiles in mouse diets.

	Founder diets			Growing diet	Mature diet
	n-3 D	n-3 N	n-3 H	AIN-93G	AIN-93M
**Fat (g/kg)**					
Lard oil	22	22	22	0	0
Sunflower oil	48	37	22	0	0
Flaxseed oil	0	7	17	0	0
Fish oil	0	4	9	0	0
Soybean oil	0	0	0	70	40
**Other nutrients (g/kg)**					
Casein	200	200	200	200	140
Corn starch	397	397	397	397	496
Maltodextrin	132	132	132	132	125
Sucrose	100	100	100	100	100
Mineral mix	35	35	35	35	35
Vitamin mix	10	10	10	10	10
Cellulose	50	50	50	50	50
Antioxidants	0.014	0.014	0.014	0.014	0.008
Choline	2.5	2.5	2.5	2.5	2.5
**Fatty acids (%)**					
ΣSFA	33.19	33.85	33.53	35.85	36.88
ΣMUFA	27.29	25.37	25.07	17.94	17.68
Σn-6 PUFAs	38.70	33.14	24.95	41.19	40.58
C18:2n-6 (LA)	38.04	32.29	24.23	41.18	40.46
C18:3n-6 (GLA)	0.41	0.47	0.34	–	0.12
C20:3n-6 (DGLA)	0.04	0.07	0.05	–	–
C20:4n-6 (AA)	0.14	0.16	0.19	–	–
C22:2n-6 (DDA)	0.05	0.09	0.07	–	–
C22:4n-6 (ADA)	0.02	0.06	0.06	–	–
C22:5n-6 (OA)	–	–	0.01	–	–
Σn-3 PUFAs	0.82	7.64	16.45	5.02	4.86
C18:3n-3 (ALA)	0.28	5.74	13.54	4.15	4.02
C18:4n-3 (STA)	0.32	0.46	0.32	0.08	0.06
C20:3n-3 (EA)	0.22	0.38	0.28	0.79	0.78
C20:5n-3 (EPA)	–	0.80	1.60	–	–
C22:5n-3 (DPA)	–	0.06	0.11	–	–
C22:6n-3 (DHA)	–	0.20	0.60	–	–
Ratio of n-6/n-3 PUFAs	47.2:1	4.3:1	1.5:1	8.2:1	8.4:1

ΣSFA, total saturated fatty acids; ΣMUFA, total monounsaturated fatty acids; Σn-6 PUFAs, total n-6 polyunsatuirated fatty acids; Σn-3 PUFAs, total n-3 polyunsaturated fatty acids; LA, linoleic acid; GLA, γ-linoleic acid; DGLA, dihomo-γ-linolenic acid; AA, arachidonic acid; DDA, decanedicarboxylic acid; ADA, adrenic acid; OA, osbond acid; ALA, α-linolenic acid; STA, stearidonicacid; EA, eicosatrienoic acid; EPA, eicosapentaenoic acid; DPA, docosapentaenoci acid; DHA, docosahexaenoic acid.

### Animals

Three- to four-week-old male C57BL/6J mice were purchased from the Gempharmatech Co., Ltd (Nanjing, China) and were housed at the animal facilities with SPF-grade condition in the National Institute of Occupational Health and Poison Control, China CDC. Following one week of recovery from transportation, the mice were randomly classified into three groups (*n* = 12 in each group) and fed with one of the n-3 D, n-3 N and n-3 H diets, respectively. All the mice were free access to water and food under the condition of a 12-h light/12-h dark cycle and cycles of air ventilation. After 12 weeks of feeding intervention, the founder male mice were mated with 10-week-old virgin female mice (1 male for 2 females per cage) and fed the AIN-93G diet (H10293G), which lasted for the pregnancy and lactation of the mating female mice. A 12-week feeding intervention for the founder male mice was set up to ensure optimal models of n-3 PUFA deficiency and supplementation, owing to that 3–6 months are needed for tissue saturation of EPA and DHA concentrations with fish oil supplementation (38–40).

After weaning at 3 weeks of age, the offspring mice from the three groups were fed the AIN-93M diet which lasted for 6 weeks. At the end of experiments, examination of the brain function (anxiety-like behaviors, depression-like behaviors, and memory) was conducted in some offspring mice (*n* = 5 in each group for the same sex). To avoid bias due to behavior tests, the other offspring mice (*n* = 8 in each group for the same sex) in a fasted state were used for determination of gene expression and DNA methylation. Each offspring mouse selected in each paternal diet group was from separate litters to avoid being born from the same father. The mice were euthanized by intraperitoneal injection of an overdose of Avertin (2,2,2-tribromoethanol) (500 mg/kg) (T-4840-2, Sigma-Aldrich Chemie GmbH, Steinheim, Germany) for anesthesia followed by decapitation. The testis, cerebral cortex and hippocampus were immediately dissected free of surrounding tissue, removed and frozen in liquid N_2_ and then transferred to −80°C for gene expression analysis. Meanwhile, tissues of the cerebral cortex and hippocampus from mice for the brain function experiment were immediately fixed by immersion in 10% paraformaldehyde and 2.5% glutaraldehyde, respectively, for immunohistochemical determination and transmission electron microscopy analysis. After mating, the founder male mice were also euthanized and testes were collected, frozen in liquid N_2_ and then transferred to −80°C for later use.

All experiments complied with the ARRIVE guidelines as well as the Guide for the Care and Use of Laboratory Animals in China. All procedures were conducted in accordance with the Animals (Scientific Procedures) 1986 Act (UK) (amended 2013) and approved by the Ethic Committee of the National Institute of Occupational Health and Poison Control, China CDC (No. EAWE-2021-06).

### Fatty acid analysis

Fatty acid analysis in diets and tissues was conducted by gas chromatography on Agilent 6890N GC equipped with a flame ionization detector (FID) and injector, using the method of fatty acid methyl esters (FAMEs). Diets and tissues of testis and brain were homogenized using a tissue disrupter in 0.9% sodium chloride solution. Preparation of FAMEs from tissue homogenates was performed according to a modified Lepage method based on our previously published (37). The quantity of each fatty acid was expressed as the percent (%) (wt/wt) of total fatty acids.

### Assessment of sperm counting and vitality

During the process of tissue collection, the cauda epididymidis of founder male mice was dissected, punctured and incubated in the prepared HEPES buffer for sperm to swim out. The supernatant was removed, centrifugated (3,000*g* for 5 min), washed twice in buffer PBS (41). The sperm preparations were assayed for sperm count and vitality assessment using the hemocytometer under the microscope.

### Behavioral experiments

Offspring mice aged 9 weeks were subjected to a series of behavioral determination. Five days before the experiment conduction, all mice were kept in the specific room to adapt to the testing environment. All tests were performed between 9 am and 5 pm. Three tests were used to determine anxiety- and depressive-like behavior. The open-field test (OFT) was conducted by placing mice in the open field (L × W × H; 50 cm × 50 cm × 30 cm) individually and allowed 5 min of free movement, and the time spent in the center area was recorded. Light/dark test (LDT) was performed according to the procedure described by Heredia (42). The rectangular box (L × W × H; 50 cm × 30 cm × 30 cm) comprised two compartments, painted black (dark compartment) and another white (light compartment), which separated by a polymeric methyl methacrylate with a centrally-positioned 7.5 × 7.5 cm opening at floor level. Mice were individually placed in the center of the dark compartment and allowed 5 min to explore the apparatus. The inter-compartmental transitions and time spent in the dark compartment were evaluated. Sucrose preference test (SPT) was performed as the described method (43). Before the test, the mice were housed in the cage with two bottles of sucrose water [2% (w/v)] to acclimate for 24 h. Then, one bottle of sucrose water was replaced by tap water for 24 h, alternating the positions of two bottles every 6 h to eliminate the possibility of side or position preference. The sucrose preference (SP) value was calculated as follows: SP (%) = sucrose intake (g)/[sucrose intake (g) + water intake (g)] × 100%.

The three-chamber test (TCT) was used for sociability assessment as described by Liu (44). Briefly, the mouse was first habituated to the empty box (L × W × H; 50 cm × 30 cm × 30 cm) with three equally sized, interconnected chambers (left, center, right) for 5 min. During the second 5-min, the tested mouse could interact either with an empty wire cup or the other wire cup contained a stranger. The time spent interacting (sniffing, crawling upon) with the two cups was recorded. Sociability index was calculated as follows: (time spent with stranger- time spent with empty cup)/(time spent with stranger + time spent with empty cup).

The novel object recognition (NOR) is an efficient method to test learning and memory in mice (45). The mouse was placed in the middle of the rectangular arena (L × W × H; 50 cm × 30 cm × 30 cm) and allowed to freely explore for 5 min and then removed out of the arena. Then two identical objects were placed in the central symmetrical positions of the arena. The mouse was again placed in the center of the arena, freely exploring the two objects for 5 min, the mouse was transported to the holding cage. One hour later, one of the training objects was replaced with a novel object, and the mouse was allowed to freely explore for 5 min. The discrimination index was expressed as the time spent exploring the novel object minus the time spent exploring the familiar object, divided by total exploration time, reflecting the preference for new objects.

### Histological examination

Immunohistochemical analysis was performed on neuron specific enolase (NSE), myelin basic protein (MBP) and glial fibrillary acidic protein (GFAP), which are specific biomarkers for neuronal cell bodies, mature myelinated oligodendrocytes and astrocytes, respectively (46–48). Formaldehyde fixed brains were treated with 70% ethanol and xylene, embedded in paraffin, sectioned at 5 mm on the coronal plane, and air dried. At the level of anterior thalamus and hippocampus, samples were taken continuously through the cerebral hemisphere. The slices were dewaxed, rehydrated in ethanol, and then incubated in 0.01 mol/L sodium citrate (pH 6.0) at 98°C for 10 minutes. After cooling, the slices were washed with 0.3% PBS Triton, incubated with 3% hydrogen peroxide for 10 minutes at room temperature, and then washed with water. Sections were incubated overnight at 4°C against antibodies for NSE (1:500) (GB11376-1), GFAP (1:1200) (GB12096), or MBP (1:200) (GB11226). After the slides were washed in PBS, incubated with goat anti-rabbit IgG HRP, and finally stained with 3,3-diaminobenzidine (DAB) (DAB chromogenic kit, G1211). Slides incubated without the addition of primary antibody were used as negative control. All antibodies and reagents were purchased from Servicebio technology Co., Ltd. (Wuhan, China). Morphometric analysis was performed on Image Pro Plus 6.0 system (media cybernetics. USA) to measure the mean optical density (OD) of NSE positive neurons, GFAP positive astrocytes and MBP positive myelinated oligodendrocytes in the cerebral cortex and hippocampus.

Structure changes in mitochondria and synapses were determined by transmission electron microscopy according to description by Rybka (49). Briefly, fresh cerebral cortex and hippocampus were fixed in glutaraldehyde (2.5%). After washed by phosphate buffer (0.1 M, pH 7.4), the slices were postfixed in 1% osmium tetroxide for 2 h. Then, they were rinsed, dehydrated, saturated and embedded in mixtures of acetone and SPI-Pon 812 resin (SPI-Chem, USA). Ultrathin slices were sectioned and poststained with uranyl acetate and lead citrate in the avoidance of carbon dioxide, and then washed with ultrapure water and dried. Imaging was done with a HT7800/HT7700 transmission electron microscope (Hitachi, Tokyo, Japan).

### Ribonucleic acid isolation and qRT-pCR

Total RNA in tissues was extracted using the RNAiso Plus (TaKaRa, Kusatsu, Japan) and complementary DNA was prepared from the total RNA using the All-in-One First-Strand cDNA Synthesis SuperMix for qPCR (OneStep gDNA Removal) (TransGen Biotech, Beijing, China) according to the procedures provided by the manufacturer. The mRNA expression of targeted genes was measured by real-time qPCR with a CFX96 Touch™ Real-Time PCR Detection System (Bio-Rad) using Top Green qPCR SuperMix (Trans Gen), with the thermocycle program consisting of an initial hot start cycle at 95°C for 30 s, followed by 40 cycles at 95°C for 5 s, 60°C for 15 s, and 72°C for 10 s. Based on their involvement in neural development, neuronal apoptosis, synaptic transmission, and neuropsychiartric disorders, a total of nine imprinted genes, Zac1, Ube3a, Peg1, Igf2 (Peg2), Peg3, Snrpn (Peg4), Ndn, Kcnk9 and RasGrf1 (34), and brain-derived neurotrophic factor (Bdnf), were included in the present study. The primer sequences can be found in Supplementary Table 1.

### Analysis of mitochondria deoxyribonucleic acid copy number

Total DNA in the offspring brain was extracted with the Animal Tissue DNA Kit (catalogue no.3101250; Simegen Biotechnology Co., Ltd.). Mitochondria DNA (mtDNA) was amplified using primers specific for the mitochondrial cytochrome oxidase subunits I (CoxI) gene. Nuclear DNA was amplified using primers specific for the 18S rRNA gene. Primer sequences can be found in Supplementary Table 1. The RT-PCR was performed on individual DNAs by using CFX96 Touch™ Real-Time PCR Detection System (Bio-Rad). The relative number of mtDNA copies (mtDNA-CN) was calculated as the normalized ratio of CoxI/18S rRNA gene.

### Deoxyribonucleic acid bisulphite conversion and sequencing

DNA methylation in differentially methylated region 1 (DMR1) of the Snrpn was determined by bisulphite sequencing. Briefly, bisulfite conversion of purified DNA of the testis and brain was treated with sodium bisulfite to convert the unmethylated cytosine into uracil using the EZ DNA Methylation Kit (catalogue no. D5002; Zymo Research). Converted DNA was amplified by nested PCR, and the PCR products were sequenced directly. The methylation fraction was calculated from the amplitude of cytosine and thymine within each CpG dinucleotide [C/(C + T)]. The assays were performed in triplicate. The primers used and annealing temperature are shown in Supplementary Table 1.

### Statistical analysis

One-way analysis of variance (ANOVA) was used to compare means in different groups using SPSS 21.0, except for body weight analysis with repeated measures ANOVA. The Kolmogorov–Smirnov test was used to evaluate whether the data is normally distributed. Following ANOVA, a *post hoc* test was conducted using either Bonferroni test or Dunnett’s T3 test for data lacking homogeneity of variance. For data with the non-normal distribution Kruskal Wallis test was used. *P* < 0.05 was considered be statistically significant in differences.

## Results

### Effects of paternal n-3 PUFA supplementation on testis fatty acid composition and sperm vitality in founder

As shown in Table 2, in founder male mice, testis DHA and total n-3 PUFAs were increased with both the n-3 N and n-3 H diet feeding, compared to the n-3 D diet feeding. Consistently, the sperm vitality was significantly increased by the n-3 N diet (84.10 ± 3.78%) and the n-3 H diet (82.02 ± 2.78%), compared with the n-3 D diet (66.62 ± 4.93%). Also, the sperm count was increased by the n-3 N and n-3 H diet (4.76 × 10^6^/L and 4.78 × 10^6^/L sperm preparations), compared to the n-3 D diet (3.51 × 10^6^/L sperm preparations).

**TABLE 2:** Fatty acid compositions of the founder testis and offspring brain.

Fatty acids (%)	Founder testis (*n* = 12)			Offspring brain (female) (*n* = 8)			Offspring brain (male) (*n* = 8)		
	n-3 D	n-3 N	n-3 H	n-3 D	n-3 N	n-3 H	n-3 D	n-3 N	n-3 H
ΣSFA	51.21 ± 7.34	45.15 ± 5.35^a^	46.00 ± 7.32^a^	56.88 ± 0.82	57.77 ± 0.81	59.47 ± 2.49	56.71 ± 2.64	55.46 ± 2.83	56.32 ± 0.99
ΣMUFA	17.27 ± 6.02	19.80 ± 6.40	18.06 ± 8.43	13.77 ± 0.50	13.60 ± 1.23	13.57 ± 0.96	13.94 ± 1.51	13.63 ± 0.91	14.21 ± 0.80
Σn-6 fatty acids	27.13 ± 3.36	24.24 ± 6.25	24.04 ± 6.18	15.52 ± 0.67	15.03 ± 1.02	14.07 ± 2.18	15.64 ± 0.78	17.48 ± 3.64	16.08 ± 0.57
C18:2n-6 (LA)	6.13 ± 3.73	6.36 ± 3.38	4.53 ± 2.80	0.94 ± 0.17	0.99 ± 0.13	0.85 ± 0.10	0.82 ± 0.07	0.91 ± 0.32	0.82 ± 0.09
C18:3n-6 (GLA)	–	–	–	0.37 ± 0.04	0.37 ± 0.15	0.37 ± 0.19	0.42 ± 0.13	0.36 ± 0.08	0.42 ± 0.08
C20:3n-6 (DGLA)	0.84 ± 0.13	1.13 ± 0.20	1.32 ± 0.34^a^	0.63 ± 0.07	0.60 ± 0.10	0.56 ± 0.15	0.64 ± 0.09	0.57 ± 0.06	0.61 ± 0.08
C20:4n-6 (AA)	9.90 ± 2.06	9.60 ± 1.90	9.91 ± 2.78	11.03 ± 0.55	10.57 ± 0.89	10.12 ± 1.38	11.11 ± 0.63	11.34 ± 0.76	11.41 ± 0.55
C22:4n-6 (ADA)	1.00 ± 0.24	0.93 ± 0.27	0.99 ± 0.28	2.13 ± 0.15	2.13 ± 0.11	2.03 ± 0.37	2.33 ± 0.14	3.58 ± 3.15	2.41 ± 0.16
C22:5n-6 (OA)	9.26 ± 2.20	7.81 ± 1.87	8.11 ± 2.23	0.44 ± 0.23	0.37 ± 0.36	0.13 ± 0.23	0.33 ± 0.34	0.71 ± 0.78	0.41 ± 0.21
Σn-3 fatty acids	4.40 ± 1.53	7.42 ± 1.54^a^	9.26 ± 1.47^a,b^	13.82 ± 0.51	13.63 ± 0.30	12.89 ± 1.02	13.71 ± 2.06	13.43 ± 1.36	13.38 ± 0.45
C18:3n-3 (ALA)	–	0.38 ± 0.28^a^	0.80 ± 0.92^a^	0.26 ± 0.04	0.25 ± 0.20	0.23 ± 0.19	0.23 ± 0.19	0.17 ± 0.12	0.30 ± 0.08
C18:4n-3 (STA)	0.05 ± 0.08	0.09 ± 0.09	0.05 ± 0.07	0.28 ± 0.03	0.24 ± 0.19	0.19 ± 0.18	0.30 ± 0.16	0.27 ± 0.07	0.31 ± 0.07
C20:3n-3 (EA)	1.74 ± 0.92	1.78 ± 0.94	1.70 ± 0.75	1.17 ± 0.22	1.49 ± 0.26	1.45 ± 0.35	1.80 ± 1.59	1.26 ± 0.62	1.16 ± 0.31
C20:5n-3 (EPA)	–	0.07 ± 0.11	0.07 ± 0.11^a^	0.22 ± 0.16	0.13 ± 0.22	0.20 ± 0.19	0.22 ± 0.22	0.21 ± 0.21	0.29 ± 0.16
C22:5n-3 (DPA)	–	–	0.04 ± 0.10^a,b^	–	0.13 ± 0.34	–	–	–	–
C22:6n-3 (DHA)	2.36 ± 0.60	4.77 ± 1.08^a^	6.16 ± 1.54^a,b^	11.89 ± 0.52	11.39 ± 0.86	10.82 ± 1.15	11.15 ± 1.09	11.52 ± 1.15	11.32 ± 0.50
C24:5n-3	0.22 ± 0.42	–	0.45 ± 0.46^ab^	–	–	–	–	–	–
Ratio of n-6/n-3 PUFAs	6.64 ± 1.63	3.26 ± 0.62^a^	2.60 ± 0.63^a,b^	1.12 ± 0.07	1.10 ± 0.07	1.10 ± 0.22	1.17 ± 0.22	1.32 ± 0.37	1.20 ± 0.06

Values are means ± SD.

^a^Compared to paternal n-3 D diet group, *P* < 0.05.

^b^Compared to paternal n-3 N diet group, *P* < 0.05.

ΣSFA, total saturated fatty acids; ΣMUFA, total monounsaturated fatty acids; Σn-6 PUFAs, total n-6 polyunsatuirated fatty acids; Σn-3 PUFAs, total n-3 polyunsaturated fatty acids; LA, linoleic acid; GLA, γ-linoleic acid; DGLA, dihomo-γ-linolenic acid; AA, arachidonic acid; DDA, decanedicarboxylic acid; ADA, adrenic acid; OA, osbond acid; ALA, α-linolenic acid; STA, stearidonicacid; EA, eicosatrienoic acid; EPA, eicosapentaenoic acid; DPA, docosapentaenoci acid; DHA, docosahexaenoic acid.

### Effects of paternal n-3 PUFA supplementation on weight and fatty acid composition of the brain in offspring

**FIGURE 1:**
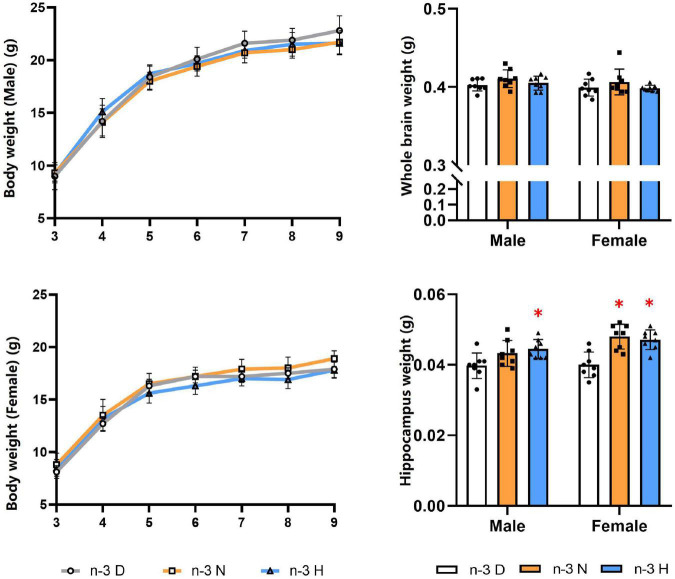
Effects of paternal n-3 PUFA supplementation on body and brain weight in offspring. Three to four-week-old male C57BL/6J mice were fed with a n-3 D, n-3 N and n-3 H diet for 12 weeks, and then mated to 10-week-old virgin female C57BL/6J mice to generate the offspring. Male and female offspring body weight were monitored weekly (*n* = 13 per diet intervention on each sex). After sacrificed at the end of experiment, brains were dissected and weighed (*n* = 8 per diet intervention on each sex). Values are means ± SD. *Compared to paternal n-3 D diet group within the same sex, *P* < 0.05; ^#^Compared to paternal n-3 N diet group within the same sex, *P* < 0.05.

As shown in Figure 1, The paternal n-3 N or n-3 H diet increased the hippocampus weight in the offspring, compared to the paternal n-3 D diet, with no effects on the body weight and the whole brain weight either in males or females. The n-3 PUFA content in the offspring brain was shown no differences among the three groups (Table 2).

### Effects of paternal n-3 PUFA supplementation on behavior and cognition in offspring

**FIGURE 2:**
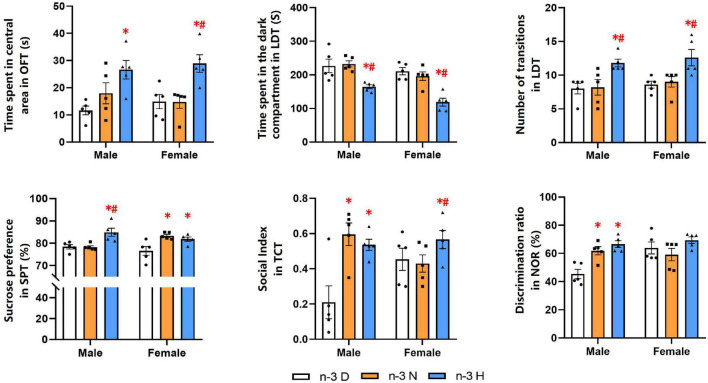
Paternal n-3 PUFA supplementation impacts behaviors and cognition in offspring. Offspring mice aged 9 weeks were subjected to a series of behavioral experiments. Time spent in central area in OFT was used to determine level of tension; Time spent in the dark compartment and the number transitions in LDT indicated anxiety behavior; Sucrose preference index in SPT was used to assess the core symptoms of depression (anhedonia); Social index in TCT represented sociability. Discrimination ratio in NOR was used to test learning and memory. Values are means ± SEM, *n* = 5 per diet intervention on each sex. *Compared to paternal n-3 D diet group within the same sex, *P* < 0.05; ^#^Compared to paternal n-3 N diet group within the same sex, *P* < 0.05.

The behavioral and cognitive experiments showed that offspring mice from the paternal n-3 H diet group had more time spent in central area in the OFT than those from the paternal n-3 D diet group. Also, they had shorter time spent in the dark section but higher number of inter-compartmental transitions in the LDT. The SPT indicated that offspring mice from the paternal n-3 N or n-3 H diet group had increased SP value. Results from the TCT exhibited that the sociability index, reflecting the length of interaction with social partner, was increased in offspring mice from the paternal n-3 N or n-3 H diet group, compared with the paternal n-3 D diet group. The changes in tests of the OFT, LDT, TCT and SPT were similar between offspring males and females. Furthermore, the NOR discrimination index was enhanced by both the paternal n-3 N diet and n-3 H diet in males instead of females in the offspring (Figure 2).

### Effects of paternal n-3 PUFA supplementation on brain histology in offspring

**FIGURE 3:**
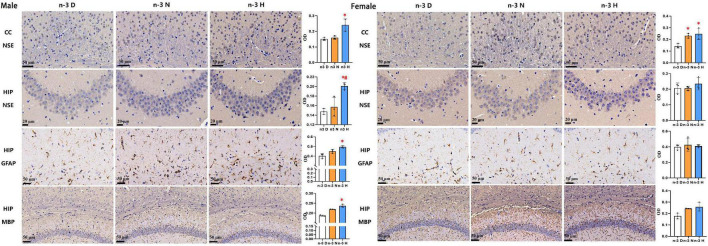
Immunohistochemical analysis in the offspring brains. The paraffin sections of the offspring cerebral cortex (CC) and hippocampus (HIP) were deparaffinized and hydrated, and specifically bound with antibodies for neuron specific enolase (NSE), myelin basic protein (MBP), glial fibrillary acidic protein (GFAP). 3,3-diaminobenzidine (DAB) staining was used to observe changes in neurons in the hippocampus (CA3 region) and cerebral cortex (cingulate), astrocytes in the hippocampus (CA1 region), and myelinated oligodendrocytes in the corpus callosum and hippocampus (CA1 region). Values are means ± SEM; *n* = 3 per diet intervention on each sex. *Compared to paternal n-3 D diet group, *P* < 0.05; ^#^Compared to paternal n-3 N diet group, *P* < 0.05.

Immunohistochemical analyses on brain NSE, GFAP and MBP in offspring were demonstrated in Figure 3. Compared to the paternal n-3 D diet group, males from the paternal n-3 H diet group had an increase in the average optical density (OD) on area of NSE-positive neurons in the cerebral cortex and hippocampus, GFAP-positive astrocytes in the hippocampus, and those from both the paternal n-3 N diet and n-3 H diet groups had more MBP-positive myelinated oligodendrocytes in the corpus callosum and hippocampus; whereas females had similar changes only in the OD on area of NSE positive neurons in the cerebral cortex, and MBP-positive myelinated oligodendrocytes in the corpus callosum and hippocampus.

**FIGURE 4:**
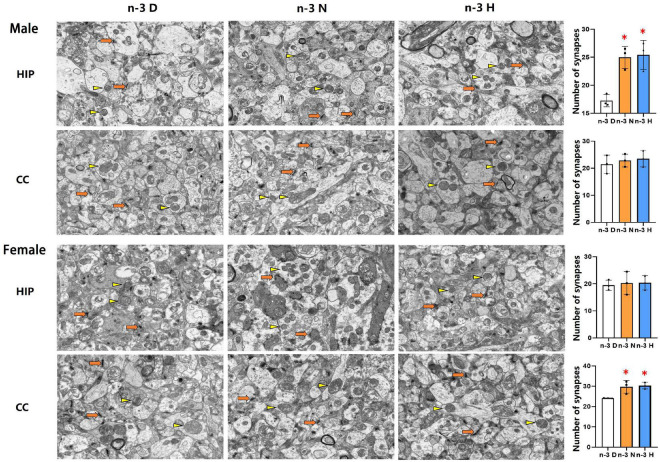
Ultrastructure changes in synapses and mitochondria in offspring cerebral cortex and hippocampus. Electron microscope photographs of the cerebral cortex (CC) and hippocampus (HIP) from offspring mice were taken at magnification (×5,000). Numbers of synapses were calculated based on electron microscope captured image. Arrows and triangles indicated synapses and mitochondria, respectively. Values are means ± SEM; *n* = 3 per diet intervention on each sex. *Compared to paternal n-3 D diet group, *P* < 0.05; ^#^Compared to paternal n-3 N diet group, *P* < 0.05.

The transmission electron microscopy demonstrated that the number of synapses was significantly increased in the hippocampus but not cerebral cortex in males from both the paternal n-3 N diet and n-3 H diet groups, compared with the paternal n-3 D diet group. In females, the synaptic number was increased in the cerebral cortex but not hippocampus in both the paternal n-3 N diet and n-3 H diet groups. No differences were observed in the ultrastructure of mitochondria in offspring hippocampus and cerebral cortex between groups (Figure 4).

### Effects of paternal n-3 PUFA supplementation on the expression of genes associated with brain function and mitochondria in offspring

**FIGURE 5:**
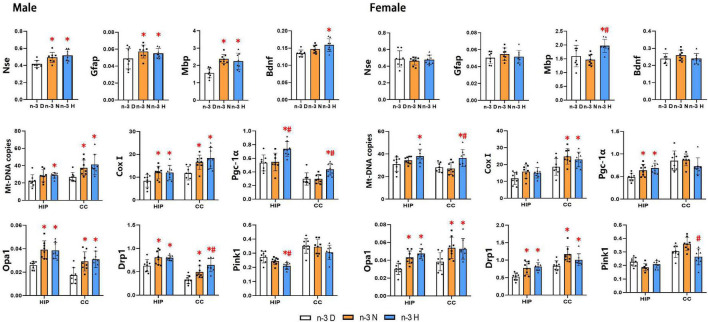
Effects of paternal n-3 PUFA supplementation on the expression of genes associated with brain function and mitochondria in offspring. RT-PCR was used to determine the mRNA expression of Nse, Gfap, Mbp and Bdnf in the hippocampus, and the mRNA expression of mitochondria associated genes (CoxI, Opa1, Drp1, Pink1, and Pgc-1α) and Mt-DNA copies both in the hippocampus (HIP) and cerebral cortex (CC) in offspring mice. The data were normalized to relative mRNA levels using the 2^–ΔCT^ method. Values are means ± SD; *n* = 8 per diet intervention on each sex. *Compared to paternal n-3 D diet group (or with in the same tissue), *P* < 0.05; ^#^Compared to paternal n-3 N diet group (or within the tissue), *P* < 0.05.

As illustrated in Figure 5, the hippocampus mRNA expression of Gfap, Mbp, Nse, and Bdnf in the male offspring was upregulated by paternal n-3 N diet or n-3 H diet, but only the Mbp expression in female offspring was upregulated by paternal n-3 H diet. Examination on mitochondria showed similar changes between the male and the female offspring, in upregulated expression of genes associated with mitochondria biogenesis (Pgc-1α, CoxI), fusion and fission (Opa1, Drp1), and downregulated expression of genes related to mitochondria autophagy (Pink1) in the hippocampus or cerebral cortex by paternal n-3 N diet or n-3 H diet. Consistently, mtDNA-CN in the hippocampus and cerebral cortex was increased by paternal n-3 N diet or n-3 H diet both in the male and female offspring.

### Effects of paternal n-3 PUFA supplementation on gene imprinting

**FIGURE 6:**
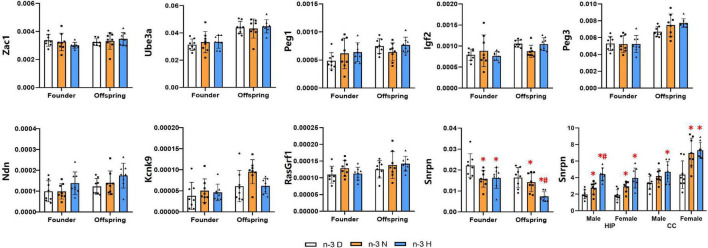
Effects of paternal n-3 PUFA supplementation on the expression of imprinted genes. RT-PCR was used to determine the mRNA expression of Zac1, Ube3a, Peg1, Igf2, Peg3, Ndn, Kcnk9, RasGrf1, and Snrpn in testes of the founders and their offspring, and Snrpn in offspring hippocampus and cerebral cortex. The data were normalized to relative mRNA levels using the 2^–ΔCT^ method. Values are means ± SD; *n* = 8 per diet intervention on each sex. *Compared to paternal n-3 D diet group within the same generation or within the same sex and tissue, *P* < 0.05; ^#^Compared to paternal n-3 N diet group within the same generation or within the same sex and tissue, *P* < 0.05.

A total of nine imprinted genes, which have been considered to be closely associated with brain development and function, were selected for expression analysis (Figure 6). The expression of Zac1, Ube3a, Peg1, Igf2, Peg3, Ndn, Kcnk9 and RasGrf1 showed no differences among the three groups, while the Snrpn was downregulated in mRNA expression by both paternal n-3 N diet and n-3 H diet in testes of the founder mice and their offspring. Therefore, the expression of Snrpn was examined in the offspring brain, and the results indicated that its expression was upregulated in the hippocampus by both paternal n-3 N diet and n-3 H diet either in males or females. In the offspring cerebral cortex, the Snrpn expression was upregulated by both paternal n-3 N diet and n-3 H diet in females, and by paternal n-3 H diet in males.

**FIGURE 7:**
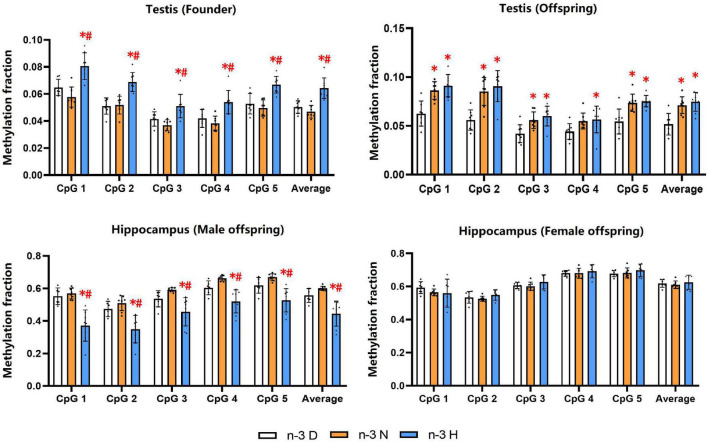
Effects of paternal n-3 PUFA supplementation on DNA methylation of imprinted gene Snrpn. Genomic DNA isolated from testes in both founders and their offspring, and the hippocampus in the offspring was given bisulfite conversion. Converted DNA was amplified by nested PCR, and the PCR products were sequenced directly for examination of CpG methylation in DMR1 of Snrpn. The methylation fraction was calculated from the cytosine and thymine within each CpG dinucleotide (C/C + T). Values are means ± SD; *n* = 6–7 per diet intervention on each sex. *Compared to paternal n-3 D diet group within the same CpG site, *P* < 0.05; ^ #^Compared to paternal n-3 N diet group within the same CpG site, *P* < 0.05.

DNA methylation analysis showed that the methylation fractions of five CpG sites of the Snrpn DMR1 in testes of the founder were increased by paternal n-3 H diet, and those in testes of the offspring were increased by paternal n-3 N diet or n-3 H diet. In the hippocampus, the methylation fractions of the Snrpn DMR1 were decreased by paternal n-3 H diet in the male offspring, with no changes in the female offspring (Figure 7).

## Discussion

A growing body of evidence suggests that the paternal diet plays a crucial role in health and disease in offspring’s adult life through epigenetic modification on sperm (50). However, the effects of paternal nutrition on offspring brain development and function are scarcely reported. In the current study, we found that paternal n-3 PUFA supplementation in preconception reduced anxiety- and depressive-like behavior (OFT, LDT, SPT), improved sociability (TCT), learning and memory (NOR) in the offspring, along with increased synaptic number, upregulated expressions of NSE, GFAP, MBP, BDNF in the hippocampus and cerebral cortex, as well as altered expressions of genes associated with mitochondria biogenesis, fusion, fission and autophagy. Furthermore, the expression of imprinted gene Snrpn was consistently downregulated in testes of the father and their offspring, and was upregulated in the cerebral cortex and hippocampus by paternal n-3 PUFA supplementation, with altered DNA methylation in DMR1 of the Snrpn. Therefore, paternal n-3 PUFA status could impact offspring brain function and histology.

As well known, adequate nutrition in early life, particularly during pregnancy and infancy, is critical in supporting healthy brain development, with long-lasting effects on cognitive, and socio-emotional skills throughout childhood and adulthood (51). Recently, the impact of paternal nutrition prior to conception on offspring brain development and function has been reported in animal models (22). Paternal methyl donor deficiency or enrichment in diets lead to alterations in offspring brain function, including consolidation-conditioned fear memory and anxiety-like behaviors (52), and hippocampal-dependent learning and memory (53). Diet-induced paternal obesity or calorie restriction during pre-conceptional period has demonstrated impairment in hippocampus-dependent learning and memory function (54), and anxiety-like behaviors in the offspring (55). Herein, our results showed that paternal n-3 PUFA supplementation reduced anxiety- and depressive-like behavior, and improved sociability, learning and memory in the offspring. Consistently, increased synaptic number and expressions of NSE, GFAP, MBP in the hippocampus and cerebral cortex in the paternal n-3 N diet and n-3 H diet groups indicated that, like maternal n-3 PUFAs (37), paternal n-3 PUFAs could promote the growth and maturation of neurons, astrocytes and myelin in the offspring.

The hippocampus is a primary region of the brain controlling the formation of memories, mood and learned behaviors. The ability to learn or form a memory requires a neuron to translate a transient signal into gene expression changes that have a long-lasting effect on synapse activity and connectivity (56). The behavioral and neurophysiological changes in offspring mice induced by paternal methyl-donor are associated with altered hippocampal expression of genes including the potassium calcium-activated channel subfamily M regulatory beta subunit 2, methionine adenosyltransferase II alpha, calcium/calmodulin-dependent protein kinase II alpha and protein phosphatase 1 catalytic subunit (52, 53). The cognitive impairment in the offspring mice caused by paternal high fat diet is attributable to the reduced expression of Bdnf (54), which is a key regulator of neural circuit development and function, mediating neuronal differentiation and growth, synapse formation, and dendritic plasticity in the mammalian brain (57). In this study, the mRNA expression of Bdnf was upregulated by paternal n-3 PUFAs in the hippocampus of male offspring, which might contribute to the memory enhancement and the improvement in expressions of Nse, Gfap, Mbp (37). As well, our previous study demonstrated that the appropriate n-3 PUFA intake during pregnancy and lactation epigeneticly affects the expression of Bdnf, and thus is beneficial for neurogenesis and anti-apoptosis in adulthood of the offspring (58).

Mitochondria play a decisive role in brain development, not only providing energy for cell proliferation and differentiation, but also determining neural stem cell differentiation and synaptogenesis (30). The upregulated expression of Pgc-1α, Cox1, Opa1 and Drp1 with downregulated expression of Pink1 by paternal n-3 PUFA supplementation indicated the increased mitochondria biogenesis, dynamics and respiratory function, with reduced autophagy. The better mitochondria function is responsible for the memory enhancement and improvement in the expression of NSE, GFAP and MBP, and conversely the mitochondria dysfunction is a hallmark of many neurological diseases, including autism spectrum disorder, hyperactivity disorder, schizophrenia, Alzheimer’s disease, Parkinson’s disease, etc. (31). Studies on other nutrients demonstrate that paternal high fat intake has significant negative effects on the embryo at a variety of key early developmental stages, with reduced mitochondria membrane potential, resulting in delayed development, reduced placental size and smaller offspring (59).

To note, no differences in the fatty acid profile in offspring brains among the three groups implied that the altered behavior and cognition by paternal n-3 PUFAs are independent of their “direct” action, which is different from maternal n-3 PUFAs. During pregnancy and lactation, maternal n-3 PUFAs are transported to the fetus and infants via the placenta and breast milk and play a directable role in maintaining proper brain development and function (4). The mechanisms by which the paternal nutrition influences the offspring’s health are poorly understood, but emerging evidence suggests that it could be transmitted through the sperm epigenome (DNA methylation, histone modifications and sncRNAs) (22, 50, 60). Paternal lifestyle and exposures to environmental factors may alter the sperm DNA methylation including imprinted genes, and consequently affect both the embryonic developmental programing and the health of future generations (47). Several studies have found that paternal fast food intake and obesity can lead to changes in DNA methylation and expression of Meg3 and Nnat in sperm (61), and Igf2/H19 in the offspring (62, 63). In this study, among the nine imprinted genes selected, just Snrpn was affected by paternal n-3 PUFA supplementation, with increased expression in offspring brains and reduced expression in testes of fathers and their offspring. Correspondently, DNA methylation fractions of the Snrpn DMR1 were increased in testes of fathers and their offspring but reduced in the hippocampus of male offspring. These suggested that the impact of paternal n-3 PUFAs on the offspring brain might be mediated by the imprinting of Snrpn, which has been found to be associated with adult neural stem cell differentiation and positively correlated with cognitive abilities in childhood (64). One mechanistic pathway has recently been identified that proper Snrpn expression directly regulates the expression of nuclear receptor Nr4a1 which is critical for cortical neurodevelopment, and that a disruption in Snrpn expression is linked to developmental brain disorders (65). In addition, the differential expression of brain mitochondrial genes was found in mouse models with partial knockout of the Snrpn promoter, and abnormal mitochondrial number and structure were found in cardiac and skeletal muscle (66). PWS-IC del mice exhibit Prader-Willi syndrome, a neurodevelopmental multifactorial genetic disorder caused by lack of Snrpn expression, including deficits in energy metabolism, behavior, cognition, and structure (67). These findings indicate the regulatory role of Snrpn in mitochondria energy metabolism. Conversely, the prominent epigenetic process, methyl groups provided by S-adenosyl methionine, in mitochondria may affect the methylation of imprinted genes (68). Thus, how Snrpn and mitochondria interact to affect brain development and function needs to be explored.

Interestingly, we found that the effects of paternal n-3 PUFA supplementation on neurobehavioral outcomes and expression of associated genes are sex-specific in the offspring. Specifically, anxiety- and depressive-like behaviors (OFT, LDT, SPT) were reduced and sociability (TCT) was improved both in offspring males and females; whereas, learning and memory (NOR) were improved only in offspring males, with paternal n-3 PUFA supplementation in preconception. Histological findings showed that the number of synapses was increased in both the hippocampus and cerebral cortex in male offspring from the paternal n-3 N diet and n-3 H diet groups, but increased only in the cerebral cortex in female offspring. In keeping with other studies, it has been demonstrated that parental environmental factors including diet, metabolism, and stress, affect the behavior and cognition of offspring differently between males and females. For example, adult female but not male offspring of dams fed the low protein diet exhibited passive, and perhaps maladaptive coping strategies in response to stress, accompanied by a marked reduction in hippocampal 5-HT1A receptor function (69). Nutrient-restricted female offspring showed improved learning, while male offspring showed impaired learning and attentional set shifting and increased impulsivity (70). Chronic consumption of a high linoleic acid diet during pregnancy, lactation and post-weaning period increases depression-like behavior in male, but not female offspring (71). With respect to the pre-conceptional paternal nutrition, as early as 20 years ago, sex-specific, male-line transgenerational responses to paternal nutrition and environment have been found in humans. Early paternal smoking is associated with greater BMI at 9 years in sons, but not daughters, and paternal grandfather’s and grandmother’s food supply was linked to the mortality risk ratios of grandsons and granddaughters, respectively (72, 73). Thereafter, male but not female offspring of fathers fed with a high protein diet exhibited increased insulin sensitivity and decreased glucose induced insulin secretion, with preserved β cell mass and plasticity following metabolic challenge (74). Whereas, when fathers were fed a high fat diet for 10 weeks before mating, female (but not male) offspring had impaired pancreatic β-cell function, with increased bodyweight and glucose intolerance, and reduced insulin secretion (75).

Although the underlying mechanisms for gender differences in parental effects on offspring are not completely known, genetics, epigenetics and gene imprinting, together with the contribution of distinct gonadal steroid hormones and associated inflammatory responses and gene expression, may be involved in this sex dimorphism (76–80). It is acceptable that the sex-specific, male-line transmissions are mediated by the sex chromosomes, X and Y (73), and that paternally expressed genes are generally growth promoting, whereas maternally expressed genes are growth restricting (81). Also, the imprinted gene Dio3 in male pups, while H19 and Xist in female pups, were upregulated by high gestational folic acid supplementation, accompanied by different expressional changes in the candidate autism susceptible gene Auts2 between male and female pups (82). Estrogen has been demonstrated to produce beneficial effects in brain development and function as well as cardioprotective effects, and the advances in understanding the structural, epigenetic and transcriptional mechanisms mediating sexual differentiation of the brain have been reviewed (83–86). It is highlighted that a gene regulatory program activated by estrogen receptor α (Erα) following the perinatal hormone surge, and sustained sex-biased gene expression and chromatin accessibility throughout the postnatal sensitive period, are of importance (86). Regarding estrogen cognitive protective effects, women with high estradiol (E2) show superior spatial reference memory (87). Female mice have a higher preference index in the NOR paradigm (62.3 ± 13.0%) than males (52.7 ± 5.9%), and are resistance to retroactive interference, which is mediated by estrogen signaling involving estrogen receptor α activation and extracellular signal-regulated kinase 1/2 in the dorsal hippocampus (88). In addition, E2 regulates hippocampus-dependent memory by promoting the synthesis of proteins and their degradation mediated by the ubiquitin proteasome system, that support structural changes at hippocampal synapses (89). As well, E2 treatment greatly upregulates the serum levels of Bdnf and transient receptor potential channels 6, the neuronal excitability indicated by an elevation in the thickness of postsynaptic density and the numbers of asymmetric synapses in rat (90). In the current study, compared to males, females in offspring from the paternal n-3 D diet group had higher NOR preference index (63.90 ± 4.30% vs 45.40 ± 3.30%), along with increased number of synapses (19.63 ± 3.42 vs 17.63 ± 3.02) and expression of Bdnf (0.24 ± 0.03 vs 0.14 ± 0.01) in the hippocampus, implying protective effects in females. Further findings showing no changes in these parameters in female offspring with paternal n-3 N diet and n-3 H diet, suggest that estrogen brain protective effects might override or mask any relationship between paternal n-3 PUFAs and offspring cognition.

The higher ratio of n-6/n-3 PUFAs (20–50:1) in modern diets, has been considered to be a risk factor for many chronic diseases (5). Individuals are required to take both series of PUFAs with the highly recommended n-6/n-3 ratio which is 4–5:1 (91). Considering the ratio of n-6/n-3 PUFAs at 1:1 in the Paleolithic diet, we previously investigated the effect of a higher intake of maternal dietary n-3 PUFAs during pregnancy and lactation on offspring and found that dietary n-6/n-3 PUFA ratio at 1–2:1 has optimal neurogenesis and maturation of neurons, astrocytes and myelin in the offspring brain (37, 58). As well, it is reported that diets with a ratio of n-6/n-3 PUFAs at 1:1 can improve the testicular development of boars and rats, and thus may more effectively reduce exogenous oxidative damage in sperm, providing a more favorable environment for sperm survival (92, 93). In the present study, the alteration in some parameters was different between the paternal n-3 N diet and n-3 H diet groups, indicating that paternal dietary n-6/n-3 PUFA ratio at 1:1 prior to conception might be more beneficial for the offspring brain development.

Our data indicate that paternal n-3 PUFAs may have an impact on offspring brain development. However, some limitations of our study should be addressed. In analyzing the impacts of paternal diet and other factors on the resultant offspring, the random effects of the mother and litter size using a random effects regression model were considered by some researchers (94–96). Unfortunately, in the current study, there exist the statistical limitations related to inability to analyze random effects form mothers and litter size due to the animal management practices in our institution, and this factor should be included in our future work. Therefore, it needs to be emphasized that the random effects statistical model is used in order to improve validity and reproducibility of research in developmental programming studies.

In conclusion, paternal pre-conceptional n-3 PUFA supplementation reduced anxiety- and depressive-like behaviors, and improved sociability, learning and memory in offspring, along with alterations in brain structural development and mitochondria, as well as the expression and DMR1 methylation of imprinted-gene Snrpn both in founder mice and their offspring. These data raise the possibility that paternal dietary factors may be relevant causal factors for mental health issues in the subsequent generation.

## Data availability statement

The original contributions presented in the study are included in the article/Supplementary material, further inquiries can be directed to the corresponding author.

## Ethics statement

The animal study was reviewed and approved by Ethics Committee of the National Institute of Occupational Health and Poison Control, China CDC (No. EAWE-2021-06).

## Author contributions

ML performed the experiment, analyzed the data and prepared the manuscript. QS carried out mouse feeding. XJ and WH performed the experiment. PL participated in the statistical analysis. XL and XF participated in designing the research. KQ designed the research and revised the manuscript. All authors read and approved the final manuscript.
