# Supplementary material for: Paternal preconceptional diet enriched with n-3 polyunsaturated fatty acids affects offspring brain function in mice
Source: Front Nutr. 2022 Oct 28;9:969848. doi: 10.3389/fnut.2022.969848 (PMC9650249; doi:10.3389/fnut.2022.969848)
Supplement: Supplementary file 1 [file Data_Sheet_1.docx]

Supplementary Material

**Table 1. Sequence of primers used for the target genes**

| Gene | Primer sequence (5′-3′) |
| --- | --- |
| **For mRNA expression** | |
| nse | F: ATCAGATCGGCTCGGTCACAG |
|  | R: AGTCCGACGACAAGATCAGCA |
| gfap | F: CCCTGGCTCGTGTGGATTT |
|  | R: GACCGATACCACTCCTCTGTC |
| mbp | F: ATCCAAGTACCTGGCCACAG |
|  | R: CCTGTCACCGCTAAAGAAGC |
| bdnf | F: GGGTCACAGCGGCAGATAAA |
|  | R: GCCTTTGGATACCGGGACTT |
| COXⅠ | F: ACTATACTACTAACAGACCG |
|  | R: GGTTCTTTTTTTCCGGAGTA |
| Opa1 | F: GGACCCAAGAGCAGTGTGTT |
|  | R: GGTTCTTCCGGACTGTGGTA |
| Drp1 | F: TGATGGGAAGGGTTATTCCA |
|  | R: TGGCCAGAGATGGGTACTTC |
| PINK1 | F: GCTTGCCAATCCCTTCTATG |
|  | R: CTCTCGCTGGAGCAGTGAC |
| PGC-1α | F: AGCCGTGACCACTGACAACGAG |
|  | R: GCTGCATGGTTCTGAGTGCTAAG |
| zac1 | F: ATGGCTCCATTCCGCTGTC |
|  | R: CTCAGCCTTCGAGCACTTGAA |
| Ube3a | F: ATCCCAGTCTGAGGACATTGA |
|  | R: GCACAAAACTCATTCGTGCAG |
| peg1 | F: GTGGTGGGTCCAAGTAGGG |
|  | R: AAGCACAACTATCTCAGGGCT |
| peg2 (Igf2) | F: GTGCTGCATCGCTGCTTAC |
|  | R: ACGTCCCTCTCGGACTTGG |
| peg3 | F: TCATGCACACTAGGGAGAACC |
|  | R: GGCAGCACTCCTACTGAAGG |
| peg4(Snrpn) | F: GGATTAGCAGGCCCTGTCAGA |
|  | R: TGCCTACAGGTGGAGGTGGA |
| Ndn | F: GAGGTCCCCGACTGTGAGAT |
|  | R: TGCAGGATTTTAGGGTCAACATC |
| Kcnk9 | F: GAGTCGGACCATGAGATGCG |
|  | R: GAGCTGATGTTGTACTTGCCT |
| RasGrf1 | F: GCCAGAAGACTTGACAACGCT |
|  | R: TCAATCTACAGGGATGGTGGAAG |
| β-actin | F: GTGACGTTGACATCCGTAAAGA |
|  | R: GCCGGACTCATCGTACTCC |
| 36B4 | F: AGATTCGGGATATGCTGTTGGC |
|  | R: TCGGGTCCTAGACCAGTGTTC |
| **For mt-DNA copy number** | |
| COXⅠ | F: CAGTCTAATGCTTACTCAGC |
|  | R: GGGCAGTTACGATAACATTG |
| 18S rRNA | F: CGCGGTTCTATTTTGTTGGT |
|  | R: AGTCGGCATCGTTTATGGTC |
| **For DNA bisulfite sequencing** | |
| Snrpn (56℃) | F out: GGTAGTTGTTTTTTGGTAGGATAT |
|  | F in: TTAGAGGGATAGAGATTTTTGTATTGTG |
|  | R out: ACTAAAATCCACAAACCCAACTAACCT |

**Table 2. Data for female founder mice**

|  | n-3 D | n-3 N | n-3 H | F | P |
| --- | --- | --- | --- | --- | --- |
| Female founder mice pre-mating weight (g) | 19.4±0.91 | 19.2±1.07 | 19.3±0.98 | 0.089 | 0.915 |
| Litter size | 60 | 89 | 72 | - | - |

Values are means ± SD, n=24 in each group.
